# Expressive Morphological Skills of Dual Language Learning and Monolingual German Children: Exploring Links to Duration of Preschool Attendance, Classroom Quality, and Classroom Composition

**DOI:** 10.3389/fpsyg.2018.00888

**Published:** 2018-06-05

**Authors:** Lilly-Marlen Bihler, Alexandru Agache, Katja Schneller, Jessica A. Willard, Birgit Leyendecker

**Affiliations:** ^1^Department of Developmental Psychology, Ruhr-University Bochum, Bochum, Germany; ^2^Department of Psychology, Heidelberg University of Education, Heidelberg, Germany

**Keywords:** bilingual, immigrant, grammar, ECEC, CLASS, plural marking

## Abstract

A growing body of research has been documenting environmental factors that support preschoolers’ vocabulary skills. However, less is known about how environmental factors are related to morphological skills of dual language learners (DLLs) and single language learners (SLLs). We examined connections with preschool experiences by investigating the effects of duration of preschool attendance, classroom quality, and classroom composition variables (percentage of DLLs and percentage of children from families with a low socio-economic status) on preschoolers’ expressive morphological skills. Several multilevel regression models were estimated using cross-sectional data from 835 children (*n* = 255 DLLs) aged 30–47 months. These children were nested in 169 preschool classrooms in Germany. As a control task, we also investigated children’s phonological processing abilities, for which we found, as expected, no differences between DLLs and SLLs. Our main finding was that DLL children scored lower in expressive morphological skills than their German monolingual peers, but this difference was considerably smaller in classrooms that scored high in instructive teacher–child interactions (measured by the Classroom Assessment Scoring System for pre-kindergarten children; CLASS Pre-K). Taken together, these results support the notion that supportive teacher–child instructive interactions have a positive impact on the development of DLLs’ morphological skills.

## Introduction

According to a German saying, language is the key to the world (Wilhelm von Humboldt). This appears to hold true. Children’s early skills in the societal language are a strong predictor of later success in school with regard to subsequent language and literacy competences, and other academic outcomes as well (Babayigit, 2014; Prevoo et al., 2016). The importance of promoting language skills at an early age is highlighted by the finding that early language-related disadvantages tend to accumulate over time (West et al., 2000; Fryer and Levitt, 2004). Fortunately, children’s language development can be supported by environmental factors (Hoff, 2006). However, studies of environmental supports for children’s language skills often focus on vocabulary, and somewhat neglect grammatical skills. This is the case despite grammatical skills holding considerable predictive power for children’s further language development (Carlisle et al., 2010; Kirby et al., 2012; Berendes et al., 2013). Grammatical skills contribute to lexical learning and reading skills, and are a prerequisite for the acquisition of the so-called cognitive-academic language proficiency (Berendes et al., 2013), which, in turn, has been found to be especially important for children’s later school success (Cummins, 2008). Our study contributes much needed information on how external environmental factors are related to children’s grammatical skills. Grammatical skills are comprised of syntax and morphology. In our study, we focus on German plural marking as a morphological skill.

While plural marking is rather straightforward in English (adding the suffix “-s” or “-es” in most of the cases), it is much more complex in the German language. Some words are the same in singular and plural (e.g., “Zimmer” can denote either *room* or *rooms*). For other words, one of many different suffixes has to be added to the root: either “-e,” “-en,” “-n,” “-s,” or “-er.” Further, some words require modifying the vowel of the word’s root with an “umlaut” (e.g., “Nagel – Nägel” [nail – nails]), and some words require adding a suffix to the root and modifying the vowel (e.g., “Baum – Bäume” [tree – trees]).

Monolingual German children start to use plurals around the age of 18 months, but with high error rates (Szagun, 2001, 2010). Three- to four-year-old normally developing German children are able to form the correct plural in about half of the cases. Five-year-old children are, on average, familiar with the rules for plural marking and make few mistakes (Clahsen et al., 1990). Thus, for monolingual German children, the acquisition of plural marking starts very early and is completed around 5 years of age. Two German studies compared immigrant and non-immigrant children’s performance in plural marking. Both studies found that immigrant children between 3 and 5 years of age scored lower in plural marking than their non-immigrant peers. The effect sizes were relatively large (Dubowy et al., 2008; Caspar and Leyendecker, 2011).

For two reasons, it is paramount to examine which environmental factors influence grammatical skills of children, both from immigrant and non-immigrant families. First, like the United States, Canada, and other countries, Germany is becoming very diverse. Today, every third child grows up in an immigrant family (OECD, 2014; Statistisches Bundesamt, 2017). Many of these children grow up as dual language learners (DLLs), exposed to two languages either from birth, or starting with enrollment into childcare. This bilingualism doubtlessly holds many personal and societal advantages. Yet, it appears to pose a challenge for many DLLs in the development of the societal language skills, which is key when laying the foundation for success in school (Castro et al., 2003; Caspar and Leyendecker, 2011; Becker and Biedinger, 2016). Second, specific environmental factors may be of greater importance for DLLs, who, on average, may receive less and later exposure to the societal language (Hoff et al., 2012; Hammer et al., 2014).

One environmental context that may be of special importance for DLLs’ grammatical skills is preschool. Even though preschool attendance rate is slightly higher for non-immigrant than for immigrant children, nearly all children between 3 and 6 years of age attend a preschool in Germany (Becker and Biedinger, 2016). While research on the relation between preschool attendance and grammar development is scarce, there is evidence from other language domains suggesting that longer preschool attendance is especially beneficial for DLLs with little German language input at home (Niklas et al., 2011; Klein and Becker, 2017). However, without also accounting for other preschool characteristics, such as classroom quality, only limited conclusions can be drawn.

With regard to classroom quality, a distinction is usually made between structural and process quality (Slot et al., 2015). Structural quality includes equipment, child–teacher ratio, as well as teacher characteristics, such as formal education. Process quality concerns the actual experiences a child has in a classroom setting (Howes et al., 2008) and is typically assessed through teacher–child interactions. Overall, there is mixed evidence concerning the influences of structural and process quality on children’s language development. Several studies in the United States could not reveal a significant effect of structural quality measures on children’s language outcomes (Early et al., 2007; Howes et al., 2008; Mashburn et al., 2008). Klein and Becker (2017) found a negative relationship when the child–teacher ratio was high, but only for Turkish immigrant children with little German language input at home.

For process quality, several studies found that instructive teacher–child interactions are modestly related to multiple language and literacy outcomes, such as receptive and expressive vocabulary, phonological awareness, rhyming, and letter naming during preschool (Mashburn et al., 2008; Burchinal et al., 2010; Wasik and Hindman, 2011; Keys et al., 2013). Emotional interactions and teacher’s classroom management behaviors are not directly related to children’s language skills (Burchinal et al., 2008; Mashburn et al., 2008). Moreover, a recent meta-analysis from Perlman et al. (2016) reports only very low and non-significant aggregated correlations between instructive interactions and different language outcomes. In all these studies, measures of process quality were obtained using the observation instrument CLASS Pre-K, which is described in more detail in the section “Materials and Methods.”

Another important feature of children’s preschool experience is the composition of their classrooms – both in terms of socioeconomic background, as well of the percentage of DLLs. Research indicates that the percentage of children from families with a lower socioeconomic status (SES) is negatively related to individual children’s language skills development (Reid and Ready, 2013; Weiland and Yoshikawa, 2014). In a German study, immigrant and non-immigrant children showed higher German language skills with a decreasing percentage of immigrant peers in their class. The effect was significantly stronger for immigrant children (Niklas et al., 2011). Thus, immigrant children’s German language development, in particular, may be supported by the exchange with children from higher SES families and from non-immigrant children, as they are likely to have more advanced German language skills.

The aim of our study was to contribute to the knowledge base on how preschool can support children’s morphological skills. Based on research showing that morphological skills are generally sensitive to environmental effects (Unsworth, 2016), we examined whether the (a) duration of preschool attendance, (b) classroom quality, and (c) classroom composition are related to expressive morphological skills of DLL and single language learner (SLL) children. We were also interested to examine whether these preschool characteristics are more closely related to DLLs’ than to SLLs’ morphological skills.

We had five research questions. First, we examined whether DLLs and SLLs morphological skills are related to children’s DLL status. We expected DLLs to score lower in morphological skills than SLLs. Second, we examined whether the quality of instructive teacher–child interactions are related to morphological skills. Measures of emotional interaction quality and teacher’s classroom management behaviors serve as control measures, because they are assumed to influence children’s motivation and engagement in learning. Thus, by controlling these measures, we were able to investigate the isolated effect of instructive interactions on children’s morphological skills. In line with previous research, we expected a positive relation for instructive teacher–child interactions, but we expected no direct associations with emotional interactions and teachers’ classroom management behaviors (Mashburn et al., 2008; Burchinal et al., 2010). Further, we expected that instructive interactions are more important for DLLs’ than for SLLs’ morphological skills, because, on average, immigrant children may receive less German language input at home and therefore may benefit more from a higher level of instructive interactions provided by the teacher. Third, we examined whether the child–teacher ratio has an impact on morphological skills. Based on results of previous studies (Dubowy et al., 2008; Howes et al., 2008; Klein and Becker, 2017), we expected no or a weak relation. Fourth, we examined the relation of duration of preschool attendance to morphological skills. Based on previous findings, we expected a positive relation, especially for the group of DLL children (Niklas et al., 2011; Klein and Becker, 2017). Fifth, we examined how measures of classroom composition, namely the percentage of children from low SES families and the percentage of DLLs in the classroom, are related to children’s morphological skills. We expected a negative relation, at least for DLLs (Niklas et al., 2011; Weiland and Yoshikawa, 2014). Beyond our five focal research questions, we examined children’s phonological processing abilities as a control measure which has been found to be unrelated to many external environmental factors (Bishop et al., 1996; Dubowy et al., 2008; Caspar and Leyendecker, 2011; Boerma et al., 2015). Here, we expected that preschool experiences are unrelated to children’s phonological processing abilities, but specifically related to children’s expressive morphological skills.

## Materials and Methods

### Sample

We used data from the first wave of an ongoing longitudinal study on language development in preschools located in North Rhine-Westphalia, the largest federal state of Germany. Several weeks prior to the start of the study, the participating preschools handed out information about the study to each parent when they brought or picked up their children. To ensure that all parents understood the procedure, the information was translated into additional languages (English, Arabic, Russian, Bulgarian, Polish, and Turkish). In addition, the preschools informed parents during parent–teacher conferences and/or posted information on their notice board. Twenty-six parents informed the preschools that they did not want their children to participate in the study. Every child received a participation certificate to show to their parents. All data were collected anonymously. Only the age, gender, age at the start of the preschool, and language spoken at home were provided by the preschool teachers. In addition, preschool teachers provided information on the percentage of children in each group whose parents’ were exempted from paying fees. We only analyzed aggregated data on larger groups of children, and not data on single children. The protocol of the study SEIKA-NRW was approved by the ethics committee of the Faculty of Psychology at the Ruhr-University Bochum.

Our sample consisted of 169 preschools classrooms in 95 public early childcare settings, and included 903 randomly selected children between 30 and 47 months of age; *n* = 803 children completed the plural marking task and *n* = 714 children completed the NWR task (see the section “Measures” description below). We asked the leading preschool teachers of each group whether they talk to the children in at least one other language apart from German every day. We received information of 139 leading preschool teachers, of which 135 answered no. The teachers of three groups spoke one additional language for less than 25% per day, and the teachers of one group for 26–50% per day. This is consistent with the percentage of 2% of bilingual preschools in whole Germany in 2014 (Frühe Mehrsprachigkeit an Kitas und Schulen e.V., 2014). Thus, German is the dominant language spoken in German preschools.

Fifty-one percent of the children were male. DLL status was operationalized based on the family language(s) spoken at home. Children who were exposed to at least one other family language apart from German (either in addition to German or solely) were considered DLLs, while monolingual German children were considered SLLs. Thirty-one percent of the children were DLLs. The DLLs in our sample spoke 36 different languages apart from German. The most frequently used languages were Turkish (31×), Russian (13×), Polish (9×), Arabic (9×), and Albanian (8×). This corresponds to the German population in which, apart from German, Turkish and Russian are the most frequently used family languages of immigrant children (Autorengruppe Bildungsberichterstattung, 2016). Additional descriptive parameters on our sample are in **Table 1**.

**Table 1:** Descriptive parameters of our sample.

Variable	*M*	*SD*	*n*
**Child level**			
Plural marking raw score	10.62	6.16	803
NWR raw score	4.83	3.14	714
Age (months)	40.37	4.65	835
Duration of preschool attendance (months)	13.38	6.87	804
**Classroom level**			
DLLs (%)	27.78	17.90	169
Children from low SES families (%)	21.54	14.62	134
Child–teacher ratio	5.55	1.73	145
Emotional support (CLASS Pre-K)	5.79	0.58	169
Classroom organization (CLASS Pre-K)	4.68	0.81	169
Instructional support (CLASS Pre-K)	2.64	0.72	169
Teacher’s age (years)	41.63	7.63	139
Teacher’s professional experience (years)	16.00	7.34	140


For children who attended the preschool for less than 6 months, we consulted with the teacher on whether the child could be tested. Children who had insufficient German language skills to understand the test instructions, children who had not yet become used to the preschool routine, and children who were too shy to be tested were excluded from the sample.

The percentage of DLL children in our sample is close to the percentage of DLLs, 36%, found in the representative German National Educational Panel Study (Olczyk et al., 2016). Our data are also representative regarding the geographical concentration of public preschools within the federal state (details on the sampling can be provided upon request). Two out of three selected preschools in our sample received additional funding from the federal state to promote children’s language development. Notably, this funding scheme was not specifically targeted for certain language promoting programs, nor did it involve using CLASS as a pedagogical enhancement tool. Also, there were no mean differences in children’s language scores and preschools CLASS ratings when comparing preschools receiving an additional funding vs. preschools which did not.

### Measures

#### Measure of Morphological Skills

Children’s morphological skills were assessed with the standardized subtest plural marking of the German *Language Development Test for 3–5-year-old children* (*SETK 3–5*; Grimm, 2001; Steinlen et al., 2010). Studies indicate the construct validity of the test for SLL and DLL children (Grimm, 2001). The test was conducted in German and analyses were performed on raw scores.

The research assistant presented the child with 10 cards, each showing (a) a single object and (b) a group of several of the same objects. The research assistant provided the term for the single object, and then asked the child to form the plural corresponding to the group of objects. Correct plural marking was scored with two points. The manual also includes a list of incorrect markings which were scored with one point. The list does not include each respective item in combination with all possible plural markers, but answers that indicate a basic understanding. For example, modfiying the root, but omitting the suffix, such as in “Büche” as the incorrect plural for “Buch” [book], instead of the correct plural “Bücher” [books] (Grimm, 2001). The rationale for this scoring is based on the stages of acquisition of the morphological rule system proposed by Grimm (2001). According to Grimm, children only memorize correct plural forms in the first stage. Children begin to understand morphological rules in the second stage. They realize that words can be separated and modified. They will make development-specific errors because regular patterns will be overgeneralized on irregular forms. In the third stage, the morphological rule system will be acquired completely. Thus, the 0–1–2 coding adequately takes into account “developmental-specific errors [overgeneralizations] as progressing development of morphological skills” (Grimm, 2001). The above example of a one point answer (“Büche” instead of “Bücher” [books]) may indicate such an overgeneralization of a pattern.

The scoring of the items was not negatively influenced by speech errors. For example, some children answered “Tühle” instead of “Stühle” [chairs]. This answer was scored with two points, because the plural was correctly marked. Dropping the initial “s” is a developmental-specific speech error at this age (Fox, 2005). Children whose answers were inaudible, and could thus not be scored, were excluded. The maximum raw score was 20 points. Internal consistency was α = 0.87 for DLLs and α = 0.84 for SLLs.

#### Measure of Phonological Processing Ability

As a control task, children’s phonological processing ability was assessed with the standardized subtest NWR of the German *Language Development Test for 3–5-year-old children* (*SETK 3–5*; Grimm, 2001; Steinlen et al., 2010). Studies indicate the construct validity of the test (Grimm, 2001). The internal consistency (Cronbach’s alpha) was α = 0.75 for DLLs and α = 0.79 for SLLs.

#### Information About the Children

Teachers completed a questionnaire with information about children’s gender (0 – male, 1 – female), age in months, duration of preschool attendance in months, and children’s family languages.

#### Compositional Variables

We used two compositional variables in our analyses. The percentage of children from low SES families in each classroom was operationalized as the percentage of children whose parents were exempted from paying a fee for their children’s preschool attendance due to their low family income. The percentage of DLLs in a classroom was based on teachers’ reports of family languages. The point-biserial correlation between these two variables was 0.50.

#### Classroom Quality

Process quality was assessed using the CLASS Pre-K (Pianta et al., 2015). The instrument captures 10 dimensions of classroom interactions, which can be assigned to three conceptually distinct domains, namely, instructional support, emotional support, and classroom organization (Pianta et al., 2015). The instructional support domain is especially important for the development of children’s language skills (Mashburn et al., 2008; Cadima et al., 2010). It is measured by three dimensions: concept development, quality of feedback, and language modeling. High values in concept development are achieved by teachers who promote children’s higher-order thinking skills. For example, the teacher frequently uses techniques to encourage children’s understanding of concepts (“What is the difference between a person and an animal?”) and provides opportunities to generate own ideas and products (if children want to make a Mother’s Day gift, the teacher helps to brainstorm the different materials which can be used and provides the material). High values in quality of feedback are achieved by teachers who use feedback to expand, and deepen knowledge (“What is the current season?” – “Spring.” – “Right. How do we know it is spring?” – “The birds are back.” – “Do all birds leave and fly south in the winter?” “…”). Teachers who give only perfunctory feedback (e.g., “yes” and “good”) score low in this dimension. Teachers who help children to learn more complex language skills score high in language modeling. For example, the teacher asks many open-ended questions that encourage children to generate and communicate their own thoughts (“Why do you think Julie is sad?”), and maps his own actions, as well as the actions of the children (“I’m going to water the plant.” or “You are taking the puppet with the red hat.”). Further, teachers introduce new vocabulary in simple terms (“What is it?” – “A cow with baby” – “Yes, it’s a cow with her calf.”).

The domain emotional support measures whether teachers provide an emotional climate, which ensures that children feel comfortable, and understood. Attachment theory posits that children explore and learn more in such environments (Hamre et al., 2013). The domain classroom organization measures whether teachers manage children’s behavior in an effective and timely manner, whether they arouse, and maintain children’s interest, and whether they provide well-prepared activities. Children in such well-organized classrooms are more engaged in learning (Hamre et al., 2013; Pianta et al., 2015).

Certified observers conducted four observation cycles of 20 min each in every classroom, using the CLASS Pre-K. All teachers present were observed. During each observational cycle, the observer rated the dimensions on a scale from 1 to 7: low (1, 2), moderate (3, 4, 5), and high (6, 7). We calculated a mean score for each dimension in each classroom from the cycles observed inside. From these mean scores, we calculated the domain scores (scale: 1–7). In addition, observers noted the number of children and teachers present during each cycle. As an indicator of structural quality, the child–teacher ratio was calculated by dividing the number of children by the number of teachers present in the classroom. On average, there were *M* = 5.55 children per teacher (*SD* = 1.73, range: 2.75–11). There were significant correlations between the child–teacher ratio and the two CLASS domains of emotional support (*r* = -0.12) and classrooms organization (*r* = -0.25). With the third CLASS domain, instructional support, the child–teacher ratio did not correlate significantly (*r* = 0.05). Reliability estimates according to Cronbach’s alphas for the three factors were α = 0.74 (emotional support), α = 0.75 (classroom organization), and α = 0.81 (instructional support).

### Analysis

#### Mean Comparisons

We investigated whether the mean scores of our examined variables differed between DLLs and SLLs. In accordance with Cohen (1988), a small effect was defined as *d* = 0.2, a medium effect as *d* = 0.5, and a large effect as *d* = 0.8.

#### Multilevel Regression Models

We estimated two sets of multilevel models, one for each outcome variable (plural marking and NWR), while NWR served only as a control task. We included only cases with no missing data on the outcome variables. We employed multilevel regression modeling, because the children in our sample were nested in classes. Following Hox (2010), our analyses were based on six steps: (1) We estimated an empty model with no explanatory variables in order to estimate the intraclass correlation coefficient (ICC). An ICC greater than zero indicates the degree to which variations in children’s outcomes are based on similarities with their classroom peers; hence, the need to account for these between-classroom variations with multilevel analysis techniques. (2) We added all variables measured on the child level with fixed slopes. (3) We then included variables measured on the classroom level. (4) Next, we added a random slope for the child level variable DLL status. (5) Finally, we added interaction terms to test whether an effect differed by DLL status, and (6) maintained significant interaction terms. All models were estimated with centered, continuous explanatory variables. For model comparison, we used the Bayesian Information Criterion (BIC). This index favors simpler models. Lower values of the BIC indicate better model fit (Brown, 2015). In our subsamples, the cluster sizes were rather small with *M* = 4.75 (plural marking subsample) and *M* = 4.29 (NWR-subsample) target children per classroom. Furthermore, around 18% (plural marking subsample) and 24% (NWR-subsample) of our clusters consisted of only one to two children. Nevertheless, we considered it appropriate to employ multilevel techniques, because a simulation study by Bell et al. (2010) indicated that an average of five observations per group is sufficient to obtain valid and reliable parameter estimates with multilevel modeling. Further, the proportion of clusters with only one child has little impact on parameter estimates, provided that a large number of clusters is used (Bell et al., 2010).

#### Missing Data

There were missing values on three independent variables: duration of preschool attendance (4%), child–teacher ratio (17%), and percentage of children from low SES families (24%). The missing values were imputed by employing multiple imputation using a two-level model. Overall, we imputed 50 datasets. Our multilevel analyses were carried out for each of the 50 imputed datasets. Parameter estimates and standard errors were averaged over the set of analyses. All analyses were conducted in Mplus Version 8. An effect was assessed as statistically significant, when *p* < 0.05.

## Results

### Mean Comparisons

In morphological skills, DLLs (*M* = 6.56, *SD* = 5.75) scored significantly lower than their German monolingual peers (*M* = 12.33, *SD* = 5.49), *t*(427.79) = 13.15, *p* < 0.01. The effect was large with *d* = 1.04 [95% confidence interval (CI) (0.88, 1.19)]. There was no significant difference between DLLs’ and SLLs’ duration of preschool attendance. Further, the classrooms attended by DLLs and SLLs did not differ significantly in the percentage of children from low SES families, in the child–teacher ratio, or in the level of observed instructional support. However, on average, DLLs did attend classrooms with lower scores on emotional support and classroom organization, as well as a higher percentage of other DLLs than SLLs. The NWR scores did not differ significantly between DLLs (*M* = 4.63, *SD* = 2.94) and SLLs (*M* = 4.92, *SD* = 3.22), *t*(463.97) = 1.21, *p* = 0.23, *d* = 0.09 [95% CI (-0.06, 0.25)]. More detailed results are presented in **Table 2**.

**Table 2:** Mean comparisons between DLLs and SLLs (*n* = 835 children).

Variable	DLLs		SLLs			
				
	*M* (*SD*)	*n*	*M* (*SD*)	*n*	*t*-Value	Cohen’s *d*
Age in months	40.49 (4.48)	255	40.31 (4.72)	580	-0.54	0.04
Duration of preschool attendance (months)	13.27 (6.67)	248	13.43 (6.96)	556	0.31	0.02
DLLs in classrooms (%)	39.51 (17.00)	255	26.93 (15.64)	580	-10.08*	0.78
Children from low SES families in classrooms (%)	22.01 (15.08)	198	19.65 (16.29)	439	-1.78	0.15
Child–teacher ratio	6.45 (2.55)	207	6.21 (2.46)	490	-1.17	0.10
Emotional support (CLASS Pre-K)	5.80 (0.54)	255	5.88 (0.56)	580	2.00*	0.15
Classroom organization (CLASS Pre-K)	4.68 (0.78)	255	4.86 (0.81)	580	3.14*	0.23
Instructional support (CLASS Pre-K)	2.51 (0.70)	255	2.53 (0.77)	580	0.37	0.03


*^∗^*p* < 0.05.*

### Multilevel Regression Analyses

#### Morphological Skills

**FIGURE 1:**
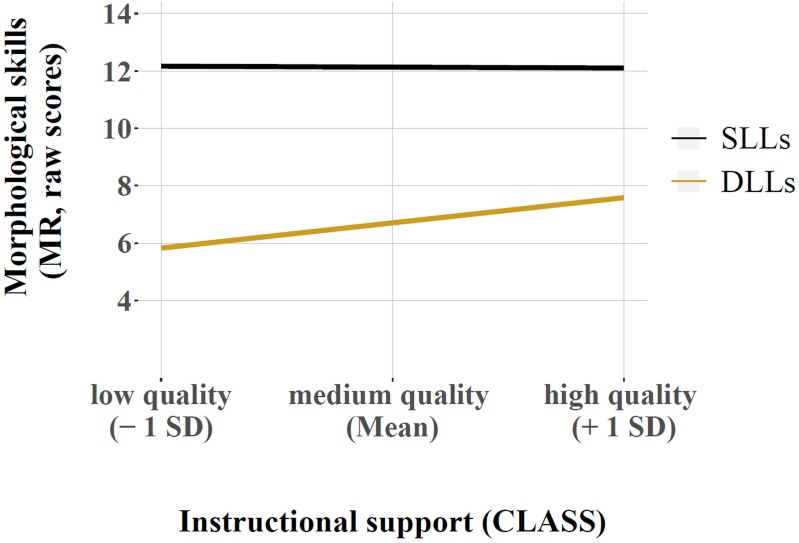
Cross-level interaction effect between children’s DLL status and observed level of instructional support on children’s morphological skills (**Table 3**, Model 6).

We obtained an intraclass correlation of ICC = 0.14, and thus, the need for multilevel modeling was indicated (Model 1). Model fit improved when we added the child level variables (Model 2), but slightly declined when we added the classroom level variables, a random slope for DLL status, and the interaction terms (Models 3–6). The ICC decreased to a value of 0.09 in Model 2, and remained stable in all subsequent models. In all models, age in months was a positive predictor, and DLL status was a negative predictor of plural marking. The duration of preschool attendance had a significant positive relation on plural marking in Models 2–4, and in Model 6. This small effect may have failed to surface in Model 5, because the level of significance was consistently just reached and overall the effect was small. With regard to our classroom composition measures, the percentage of children from low SES families had a small significant negative effect on plural marking in all models. Besides these direct effects, our analyses revealed a positive significant interaction effect between DLL status and instructional support. To better understand the pattern of this cross-level interaction effect, we plotted simple slopes at low (*M* -1 SD), mean, and high levels (*M* +1 SD) of instructional support (**Figure 1**), using the equations provided by Bauer and Curran (2005). The estimated difference in plural marking scores between DLLs and SLLs was 4.52 points (*p* < 0.01) for children attending high-quality classrooms (mean instructional support sample value +1 SD), but it was 6.34 points (*p* < 0.01) for children attending low-quality classrooms (mean value -1 SD). For acquiring morphological skills, the level of instructional support is predictive only for DLLs, but not for SLLs. Detailed results are presented in **Table 3**.

**Table 3:** Results of multilevel regression analyses for predicting plural marking (raw scores).

Variable	Model 1: Intercept only	Model 2: Child level predictors	Model 3: Classroom level predictors	Model 4: Random slope	Model 5: Interactions	Model 6: Only significant interactions
Intercept	10.59 (0.28)*	12.21 (0.31)*	12.13 (0.30)*	12.13 (0.30)*	12.08 (0.30)*	12.14 (0.30)*
Gender (male = 0, female = 1)		0.02 (0.39)	-0.03 (0.39)	-0.01 (0.39)	0.03 (0.38)	0.01 (0.39)
DLL status (SLL = 0, DLL = 1)		-5.55 (0.47)*	-5.34 (0.49)*	-5.43 (0.50)*	-5.53 (0.52)*	-5.45 (0.49)*
Age in months		0.30 (0.05)*	0.31 (0.05)*	0.31 (0.04)*	0.32 (0.04)*	0.32 (0.04)*
Duration of preschool attendance (months)		0.09 (0.04)*	0.09 (0.04)*	0.09 (0.04)*	0.06 (0.04)	0.09 (0.04)*
DLLs in classrooms (%)			-0.02 (0.02)	-0.02 (0.02)	-0.03 (0.02)	-0.02 (0.02)
Children from low SES families (%)			-0.04 (0.02)*	-0.04 (0.02)*	-0.04 (0.02)	-0.04 (0.02)*
Child–teacher ratio			-0.12 (0.11)	-0.12 (0.11)	-0.09 (0.12)	-0.12 (0.11)
Emotional support (CLASS Pre-K)			-0.13 (0.57)	-0.15 (0.57)	-0.13 (0.40)	-0.17 (0.57)
Classroom organization (CLASS Pre-K)			0.06 (0.42)	0.11 (0.41)	0.13 (0.40)	0.16 (0.40)
Instructional support (CLASS Pre-K)			0.32 (0.30)	0.20 (0.29)	-0.04 (0.31)	-0.04 (0.32)
DLL status × duration of attendance					0.09 (0.07)	
DLL status × instructional support					1.37 (0.60)*	1.27 (0.61)*
DLL status × DLLs (%)					0.03 (0.03)	
DLL status × low SES families (%)					0.01 (0.04)	
DLL status × child–teacher ratio					-0.12 (0.19)	
*σ*^2^_e_ (within)	32.48 (1.43)*	24.72 (1.17)*	24.77 (1.18)*	23.82 (1.37)*	23.84 (1.39)*	23.83 (1.37)*
*σ*^2^_u0_ (between)	5.41 (1.28)*	3.35 (1.08)*	2.46 (0.96)*	1.88 (1.11)	1.83 (1.10)	1.88 (1.10)
*σ*^2^_u1_ (DLL status)				5.05 (3.35)	3.44 (3.22)	4.11 (3.22)
BIC	5187	4981	5005	5013	5038	5016


*Numbers in the table are coefficients from multilevel regression analyses. Standard errors are provided in paranthese. L1, measured on the child level; L2, measured on the classroom level. ^∗^*p* < 0.05.*

#### Non-word Repetition

Based on the variance estimates from an intercept-only model, with no explanatory variables (Model 1), the intraclass correlation ICC was 0.08. This supports the application of multilevel modeling. Model fit improved when we added the child level variables (Model 2), but the addition of the classroom level variables did not further improve the fit. In Model 2, the ICC increased to 0.10 and remained stable in the subsequent models. This suggests that the between classroom variance in NWR scores was not accounted for by our predicators. In all models, the only variable with a significant effect was age in months. Detailed results are presented in **Table 4**.

**Table 4:** Results of multilevel regression analyses for predicting NWR (raw scores).

Variable	Model 1: Intercept only	Model 2: Child level predictors	Model 3: Classroom level predictors	Model 4: Random slope	Model 5: Interactions
Intercept	4.82 (0.14)*	4.84 (0.20)*	4.82 (0.20)*	4.83 (0.20)*	4.84 (0.20)*
Gender (male = 0, female = 1)		0.08 (0.25)	0.08 (0.24)	0.06 (0.24)	0.04 (0.24)
DLL status (SLL = 0, DLL = 1)		-0.28 (0.26)	-0.24 (0.27)	-0.23 (0.27)	-0.25 (0.27)
Age in months		0.23 (0.02)*	0.23 (0.02)*	0.23 (0.02)*	0.23 (0.02)*
Duration of preschool attendance (months)		0.04 (0.02)	0.04 (0.02)	0.04 (0.02)	0.03 (0.02)
DLLs in classrooms (%)			-0.01 (0.01)	-0.01 (0.01)	-0.01 (0.01)
Children from low SES families (%)			0.01 (0.01)	0.00 (0.01)	0.02 (0.01)
Child–teacher ratio			0.05 (0.06)	0.05 (0.06)	0.05 (0.08)
Emotional support (CLASS Pre-K)			0.17 (0.25)	0.17 (0.26)	0.14 (0.26)
Classroom organization (CLASS Pre-K)			-0.35 (0.23)	-0.34 (0.23)	-0.33 (0.23)
Instructional support (CLASS Pre-K)			-0.33 (0.18)	-0.32 (0.19)	-0.39 (0.21)
DLL status × duration of attendance					0.02 (0.03)
DLL status × instructional support					0.26 (0.33)
DLL status × DLLs (%)					0.01 (0.02)
DLL status × low SES families (%)					-0.04 (0.02)
DLL status × child–teacher ratio					-0.01 (0.13)
*σ*^2^_e_ (within)	9.05 (0.47)*	7.60 (0.39)*	7.61 (0.40)*	7.31 (0.44)*	7.30 (0.44)*
*σ*^2^_u0_ (between)	0.81 (0.34)*	0.97 (0.35)*	0.77 (0.35)*	1.23 (0.50)*	1.27 (0.51)*
*σ*^2^_u1_ (DLL status)				1.28 (1.31)	0.84 (1.26)
BIC	3671	3590	3618	3629	3656


*Numbers in the table are coefficients from multilevel regression analyses. Standard errors are provided in parantheses. L1, measured on the child level; L2, measured on the classroom level. ^∗^*p* < 0.05.*

## Discussion

Even though much is known about environmental effects on preschool children’s vocabulary skills, research on morphological skills is rare – particularly on those of DLL children. However, this knowledge is important to successfully promote children’s school readiness. We addressed this gap in the literature by investigating how DLLs’ and SLLs’ duration of preschool attendance, the composition of their classrooms, and the quality of their classrooms are related to their morphological skills. Further, we investigated whether these relations are moderated by DLL status. Our main finding was that DLLs scored lower in expressive morphological skills than SLLs. However, this difference was smaller in classrooms that scored high in instructive teacher–child interactions.

### Morphological Skills of DLLs and SLLs

Morphological skills were substantially related to children’s language background. Controlling for the duration of preschool attendance, classroom composition, and classroom quality, SLLs scored, on average, 5.45 points higher than DLLs which corresponds to a difference of nearly 1 SD. This effect cannot be attributed to age differences between DLLs and SLLs (**Table 2**), but indicates that the development of morphological skills is less proficient in DLLs. In comparison to SLLs, DLLs were more likely to receive only one point instead of two point scores for each of the 10 items. This indicates that, on average, the DLLs were at a different point in their development of morphological skills. Hammer et al. (2014) showed that even though DLLs required more time to develop solid grammar skills in the societal language, they tend to catch up over time. Other studies found that a low performance in morphological skills can negatively affect the performance in other academic measures (Carlisle et al., 2010; Kirby et al., 2012). Thus, whether this early difference in morphological skills has lasting consequences for the development of other language skills (e.g., vocabulary), or literacy skills, may be investigated in further studies.

### Classroom Process Quality and Morphological Skills

As expected, the quality of the classroom emotional interactions and classroom management behaviors were not related to average levels of children’s morphological skills. Instructive interactions, however, were related to DLLs’ morphological skills. Our results indicated that SLLs did not benefit from a higher level of instructive interactions. The most straightforward explanation is that the SLLs received sufficient quality of German language input at home to develop morphological skills. The finding that DLLs morphological skills were related to the level of instructive interactions implies its potential for promoting their morphological skills. The average level of instructional support was low in our sample, and thus, there is a considerable room for improvement. The low average level of instructional support found in our sample is in line with other results from Germany (von Suchodoletz et al., 2014; Stuck et al., 2016) and the United States (Hamre et al., 2014). Further studies may reveal what specific features of instructional support are supportive of DLL’s morphological sills, and whether the level of instructional support is also positively related to other language or literacy domains of DLLs.

### Child–Teacher Ratio and Morphological Skills

The child–teacher ratio was not related to children’s competence in morphological skills, and there was no interaction effect with DLL status. Our result is in line with previous research which indicated that process quality is more important for children’s language development than characteristics of structural quality (Howes et al., 2008). Our results, however, are based on cross-sectional data. Therefore, we cannot rule out that variations in child–teacher ratio may be important for children’s morphological skills. One alternative explanation for our finding is that the effect may be mediated by another variable. For example, a lower child–teacher ratio may result in higher ratings of process quality, and this may result in a better performance of DLL’s in morphological skills (Nichd, 2002). Investigating whether such “hidden” mediation mechanisms are in place may be a subject for future longitudinal analyses.

### Duration of Preschool Attendance and Morphological Skills

We expected longer preschool attendance to be positively related to morphological skills, at least for DLLs, and we found a positive significant effect for all children. Why does this effect apply for DLLs as well as SLLs? An explanation might be that attending a preschool has a positive effect on the overall quantity of German language input. Preschools provide many opportunities for interacting with a large group of peers and with several teachers, or to listen to others’ conversations. Blom et al. (2012) found that variations in input quantity can impact children’s morphological skills. However, even though the effect was significant, it was low in size. Compared to the somewhat stronger relation of the level of instructive interactions on DLLs’ morphological skills, this might indicate that for DLLs, the quality of the input of the German language is more important than the mere quantity.

### Classroom Composition and Morphological Skills

Our results indicated that, on average, children from classrooms with higher percentages of low SES families scored slightly lower in their morphological skills. This result has to be interpreted carefully, because we had no information on the individual child’s family SES. Therefore, the composition effect may have surfaced because children in classrooms with a higher number of children from low SES families may have a higher probability of coming from a low SES family themselves. On average, children from low SES families tend not to perform as well on language measures as children from high SES families (Rowe and Goldin-Meadow, 2009; Becker, 2010; Fernald et al., 2013).

Unexpectedly, our second measure of classroom composition, the percentage of DLLs in the classroom, was unrelated to DLLs’ and SLLs’ morphological skills. This contradicts previous multilevel cross-sectional results from other language domains (Niklas et al., 2011). One explanation for these divergent findings might be that we simultaneously investigated the effects of duration of attendance, classroom composition, and classroom quality on morphological skills, while the previous study did not include classroom quality in their analyses. Thus, our results suggest that instructive teacher–child interactions are more important for the development of morphological competencies than a classroom’s language composition.

### Phonological Processing Abilities

In line with our expectations, children’s phonological processing abilities were not related to children’s language background. Thus, our results concur with the current state of research, by indicating that performance in NWR is fairly independent of the language(s) spoken at home (Boerma et al., 2015). Further, children’s phonological processing abilities were not contingent on our examined child level and classroom level variables. This indicates that NWR is also independent from preschool characteristics, such as classroom composition and classroom quality. The independence from preschool factors and children’s DLL status may be a valuable feature for diagnostic purposes.

### Limitations

The results of this study are based on cross-sectional data. Hence, the direction of causality is unclear and conclusions should be drawn with caution. Nevertheless, one strength of this study is the comparatively large sample size.

A more detailed differentiation of DLLs’ language background and input would have been desirable. The effects of environmental factors on morphological skills in the societal language may vary depending on the amount of societal language input DLLs receive at home. For example, in one study, longer preschool attendance had only a significant effect on the expressive vocabulary of those DLLs who received little German language input at home (Klein and Becker, 2017). Furthermore, we could not distinguish between DLLs’ ages of acquisition of the German language, which has been found to have an important impact on DLLs’ language skills (Paradis et al., 2013; Hammer et al., 2014). Similarly, it would have been desirable to distinguish between DLLs who speak a family language that has plural marking similar to German and DLLs who do not.

Another limitation concerns the lack of family level covariates, especially for the individual child’s family SES and for the home literacy environment. Studies on other language domains illustrate that the home environment has a major influence on language development (Crosnoe et al., 2010; Unsworth, 2016). Therefore, larger samples that allow the simultaneous estimation of family and preschool variables would be desirable.

### Conclusion and Implications

The purpose of this study was to examine the effects of duration of preschool attendance, classroom composition, and classroom quality on children’s language skills in relation to their DLL status. We found that children who attended preschool longer scored higher in expressive morphological skills. This suggests an advantage for children who are enrolled in school at an early age. We can assume that children’s morphological skills improve by talking with, or listening to, peers and teachers. Our main implication resides in the finding that DLLs in classrooms with higher instructive quality scored better in morphological skills. Therefore, it can be recommended to foster teachers’ competences in providing instructive interactions for this group of children.

In Germany, there is an ongoing public debate whether children’s language skills develop better in preschool classrooms with few DLLs and few children from low SES families. As a result, some parents use the composition of the preschool classroom as a criterion for selecting a preschool for their child. We found that the effect of classroom composition variables disappeared, or was diminished (in the case of SES), once the individual language status was adjusted for. This suggests that parents may base their decision for a preschool on a feature that might not have the crucial impact they attribute to it. In addition, it is difficult for parents to assess classroom interaction quality (Cryer et al., 2002). Thus, policymakers bear part of the responsibility in ensuring that children have access to preschools that provide a stimulating learning environment. This may be done by continuously monitoring and promoting interaction quality of “all” classrooms. Trainings of interaction quality may include the promotion of students’ higher-order learning skills, the usage of feedback to expand and deepen knowledge, and supporting children in learning more complex language skills.

## Ethics Statement

This study was carried out in accordance with the recommendations for psychological research of the Deutsche Gesellschaft für Psychologie (DGPs; German Psychological Society). All parents received detailed, written information prior to the start of the study from the preschool teachers. To ensure that all parents understand the procedure, they received this information in their preferred language. Parents who did not want their children to participate in the study were asked to inform the preschool teachers. Only children of parents who did not disagree with their children’s participation were included in the study. All data were collected anonymously. Only the age, gender, age at the start of the preschool, and language spoken at home was provided by the preschool teachers. In addition, preschool teachers provided information on the percentage of children whose parents were exempted from paying fees. This information was provided only for the entire group and not for individual children. The protocol was approved by the ethics committee of the Faculty of Psychology at the Ruhr-University Bochum.

## Author Contributions

All authors designed the study and planned the data collection. AA conceived the original idea and conducted preliminary multilevel analyses. L-MB conducted the statistical analyses and drafted the manuscript. BL supervised the project. All authors were involved in writing the manuscript, discussed the results, and approved the submitted version.

## Conflict of Interest Statement

The authors declare that the research was conducted in the absence of any commercial or financial relationships that could be construed as a potential conflict of interest.
